# Spatiotemporal Characteristics of Particulate Matter and Dry Deposition Flux in the Cuihu Wetland of Beijing

**DOI:** 10.1371/journal.pone.0158616

**Published:** 2016-07-20

**Authors:** Lijuan Zhu, Jiakai Liu, Ling Cong, Wenmei Ma, Wu Ma, Zhenming Zhang

**Affiliations:** 1 College of Nature Conservation, Beijing Forestry University, Beijing, China; 2 School of Natural resources, West Virginia University, Morgantown, West Virginia, United States of America; NSYSU, TAIWAN

## Abstract

In recent years, the rapid development of industrialization and urbanization has caused serious environmental pollution, especially particulate pollution. As the “Earth’s kidneys,” wetland plays a significant role in improving the environmental quality and adjusting the climate. To study how wetlands work in this aspect, from the early autumn of 2014 to 2015, we implemented a study to measure the PM concentration and chemical composition at three heights (1.5, 6, and 10 m) during different periods (dry, normal water, and wet periods) in the Cuihu wetland park in Beijing for analyzing the dry deposition flux and the effect of meteorological factors on the concentration. Results indicated that (1) the diurnal variations of the PM2.5 and PM10 concentrations at the three heights were similar in that the highest concentration occurred at night and the lowest occurred at noon, and the daytime concentration was lower than that at night; (2) the PM2.5 and PM10 concentrations also varied between different periods that wet period > normal water period > wet period, and the concentration at different heights during different periods varied. In general, the lowest concentration occurred at 10 m during the dry and normal water periods, and the highest concentration occurred at 1.5 m during the wet period. (3) SO_4_^2−^, NO_3_^−^, and Cl^−^ are the dominant constituents of PM2.5, accounting for 42.22, 12.6, and 21.56%, respectively; (4) the dry depositions of PM2.5 and PM10 at 10 m were higher than those at 6 m, and the deposition during the dry period was higher than those during the wet and normal water periods. In addition, the deposition during the night-time was higher than that during the daytime. Moreover, meteorological factors affected the deposition, the temperature and wind speed being negatively correlated with the deposition flux and the humidity being positively correlated. (5) The PM10 and PM2.5 concentrations were influenced by meteorological factors. The PM2.5 and PM10 concentrations were negatively correlated with temperature and wind speed but positively correlated with relative humidity.

## Introduction

Atmospheric particulate matter is a mixture of small particles and liquid droplets suspended in the atmosphere, which can influence the air quality, affecting the atmospheric visibility. Some researchers also indicated that particulate matter pollution can be detrimental to human health [1–4], especially PM10 and PM2.5 (particulate matter with aerodynamic equivalent diameters less than 10 and 2.5 μm, respectively). They have a chronic effect on the human body, damaging somatic functions, causing coughing and other airway problems, and leading to permanent damage to the lungs and emphysema [5–8].

According to prior data, which was taken seriously by governments and the public, about 3.2 million people died of air pollution in 2010, and 1.2 million were from China [9]. Along with the rapid development of the economy and urbanization, China is suffering from severe air pollution, especially in Beijing, Shanghai, and Guangzhou. The annual mean concentration of particulate matter with an aerodynamic equivalent diameter equal to or less than 2.5 μm was 100 μg/m^3^ in Beijing [10], which greatly exceeded the China Ambient Air Quality Standard (BG3095-12), grade II standard. It is therefore imperative that China’s government alleviates air pollution.

Wetlands, as “the Earth’s kidneys,” have a significant effect on improving air quality; many researchers have studied the function of wetlands in alleviating air pollution and have indicated that this can increase the relative humidity and thus reduce the concentration of particulate matter. An assessment of the particulate matter of a wetland in Beijing showed that the PM concentration was highest from 6:00–9:00 and at its lowest from 15:00–18:00, and that the annual average concentration variation was highest during the dry period and lowest during the wet period [11]. Also, research on the calculated and measured dry deposition of particulate matter on different surfaces (urban forest, wetland, and lake) in the Beijing Olympic Park showed that the deposition velocity on the wetland was lower than on the forest canopy, and that the forest could collect more PM than the wetland [12]. A study of the removal efficiency of particulate matter for different underlying surfaces (bare land, urban forest, and lake) found that the forest has the highest efficiency with respect to removing particles [13]. A study of Hengshui lake wetland, using the particle measurement system (PMS) to study the aerosol concentration, indicated that the aerosol concentration over the wetland is lower than in the city [14]. An analysis of the soil, sediment, and atmospheric samples to study the origin of polycyclic aromatic hydrocarbons (PAHs) indicated that the concentration of PAHs is 82.45 × 10^−9^ ng/m^3^, and that this mainly originates from the atmosphere [15]. However, these studies only focused on the particulate matter concentration in the wetland or compared the deposition velocity in the wetland with other different surfaces; they ignored the spatiotemporal variation of the PM concentration and the chemical composition and deposition in the wetland, which are important in understanding the mechanism by which particulate matter in the wetland is reduced.

The aims of this study are as follows: (1) analyzing the spatiotemporal variation of the PM concentration; (2) analyzing the spatiotemporal variation of the PM2.5 chemical composition; (3) calculating the dry deposition flux of PM at different heights during different periods; and (4) analyzing the influence of meteorological factors on the PM concentration.

## Materials and Methods

### Study area

Cuihu Wetland Park (area: 1.57 km^2^, of which about 0.09 km^2^ is water; length: 1.9 km; width: 1.2 km) is managed by Beijing Municipal Bureau of Landscape and Forestry. It is located to the north of the Shangzhuang reservoir in the Haidian District of Beijing.

**Fig 1:**
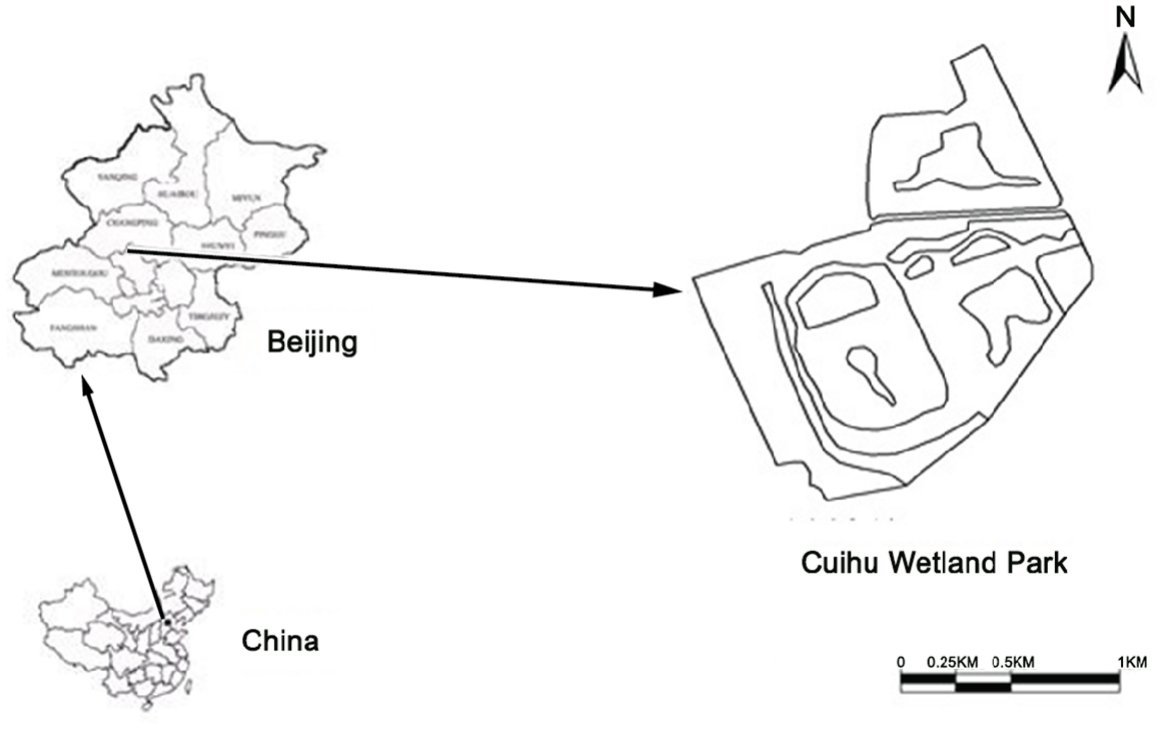
The location of Cuihu Wetland Park.

The climate is characterized by dry and strong winds with dust in spring (March–June). In the summer (June–September), the weather is stable and hot and is dominated by precipitation. The autumn (September–December) is sunny and characterized by moderate temperatures. The winter is cold and dry. The location of Cuihu Wetland Park is shown in Fig 1.

### Experimental design

#### Ethics statement

This study did not involve endangered or protected species.

#### Sample collection

**Fig 2:**
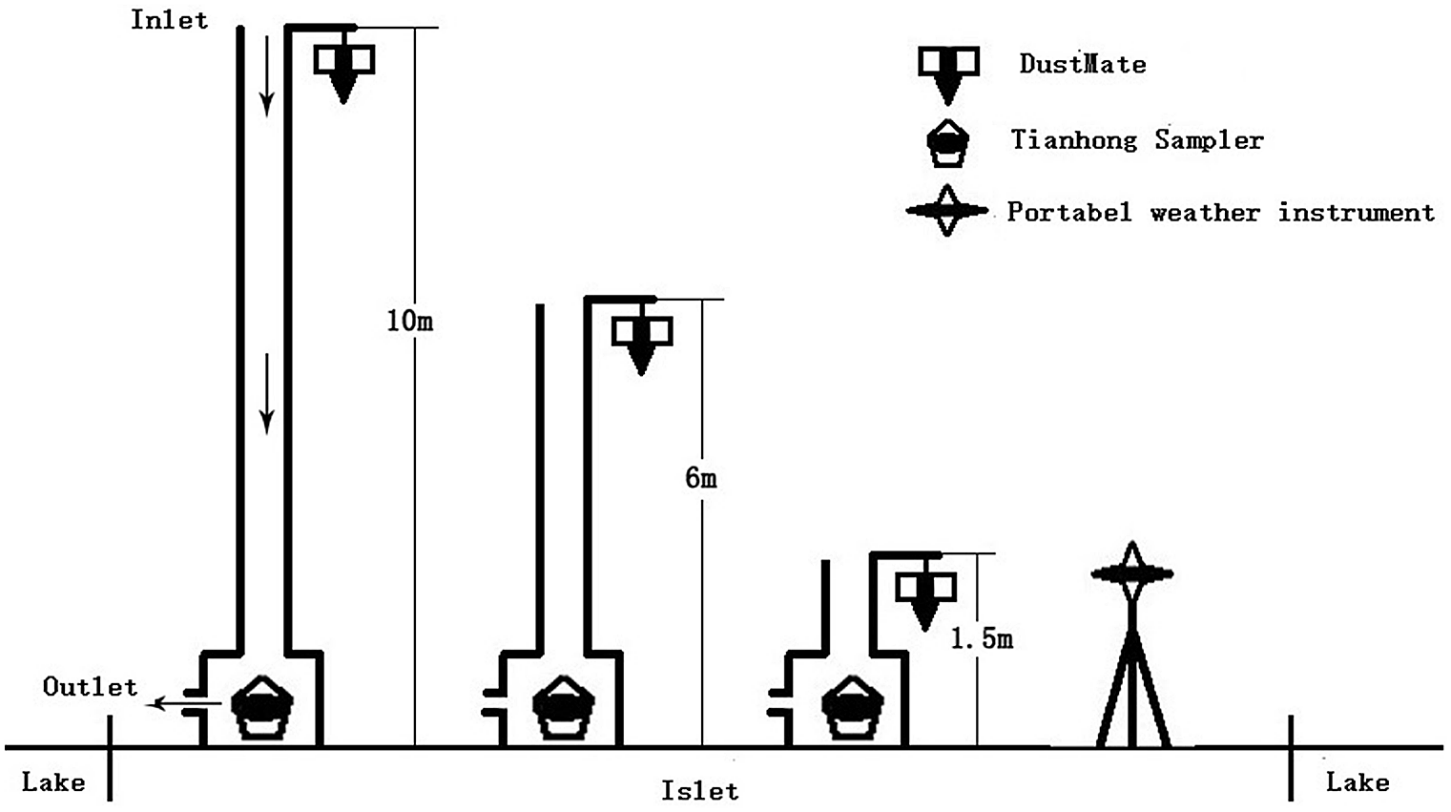
The design of the experimental instruments.

As shown in Fig 2, three heights (1.5, 6, and 10 m) were used in this study. A handheld Dust Mate particulate matter sampler (DUSTMATE, Turnkey Instruments Ltd., United Kingdom), Tianhong samplers (TH-150C, Westernization Instrument Technology Co., Ltd., Beijing, China), and portable meteorological instruments (Kestrel 4000 Pocket Weather Meter, Nielsen-Kellerman, Boothwyn, PA, USA) were deployed at each height to monitor the PM2.5 and PM10 concentrations, the composition, and meteorological factors (temperature, relative humidity, and wind speed). The Dust Mate recorded PM2.5 and PM10 concentration data every 5 minutes from 7:00 am to the next 7:00 am in experimental periods, and the meteorological instruments recorded the meteorological data every minute. Tianhong samplers collected samples at a flow rate of 120 L min^−1^. The procedure for using the above instruments to collect the PM concentration and chemical composition was given in previous studies [16] and some researchers have successfully used this method [17]. Also, the statistical analysis methods applied in this paper have been derived from previous research based on this experimental design [18–19] and have been used in previous studies [12].

#### Ion analysis

To analyze the chemical composition, one fourth of each sample was cut into pieces and dissolved in 50 ml of deionized water. The anions and cations were then analyzed by ion chromatography (IC, WAYEE, 6200) using a separation column (IC SI-52 4E 4 mmlD*25cm for anions and TSKgel Super IC-CR 4.6 mmlID*15cm for cations).

### Data analysis

#### Period division

According to the amount of water in the wetland, we can divide the whole year into three periods: the normal water period (spring and autumn), the wet period (summer), and the dry period (winter).

### Calculation of dry deposition flux

The dry deposition flux can be calculated as follows [18–19]:

$$F=v_{d}\cdot \Delta c$$ (1)

Where *F* is the deposition flux, Δ*c* is the concentration difference between the constant-flux layer and the deposition layer, and *v*_*d*_ is the deposition velocity, which can be defined as follows:

$$\frac{1}{v_{d}}=\frac{1}{V_{C}}+\frac{1}{V_{D}}-\frac{V_{g}}{V_{C}\cdot V_{D}}$$ (2)

where *V*_*g*_ is the gravitational settling speed based on the dry particle diameters, *V*_*C*_ is the total transfer velocity in the constant-flux layer, and *V*_*D*_ is the total transfer velocity in the dry deposition layer. These can be calculated as follows:

Vg=Pp⋅Cc⋅dp2⋅g/18⋅μa (3)

$$V_{C}=V_{C}^{\prime }+V_{g}$$ (4)

$$V_{D}=V_{D}^{\prime }+V_{g}$$ (5)

where *C*_*c*_ is the Cunningham correction factor, *P*_*p*_ is the density of the particles and can be replaced by the particle concentration, *d*_*p*_ is the particle diameter, *μ*_*α*_ is the air dynamic viscosity, *V*_*C*_′ is the transfer velocity without gravity in the constant-flux layer, and *V*_*D*_′ is the transfer velocity without gravity in the dry deposition layer. These can be calculated as follows:

$$C_{c}=1+\frac{2\lambda }{d_{p}}\cdot (1.257+o.4e^{-0.55\cdot d_{p}/\lambda })$$ (6)

$$V_{C}^{\prime }=\frac{1}{1-k}\cdot C_{d}\cdot \text{u}(z)$$ (7)

$$V_{D}^{\prime }=-\alpha \cdot m+k^{-1}\cdot C_{d}\cdot u(z)\cdot Sc^{-1/2}+10^{-3}\cdot St$$ (8)

where *λ* is the mean free path of air (65 nm), *α* is constant (10^3^*cm* ⋅ *s*^−1^ / (1*g cm*^−2^ ⋅ *s*^−1^)), *C*_*d*_ is the drag coefficient, *C*_*d*_ = [(1.3 ± 0.3) × 10^−3^], *S*_*c*_ is the Schmidt number, and *S*_*t*_ is the Stokes number, which can be calculated as

$$Sc=\frac{v_{a}}{D_{B}}=\frac{\mu_{a}}{P_{p}}$$ (9)

$$S_{t}=\tau_{p}\cdot u(z)/d_{n}$$ (10)

where *d*_*n*_ is the dimension of the vegetation element for wetland, usually (*d*_*n*_ = 1) [20], and *τ*_*p*_ is the particle relaxation time, which can be presented as

$$\tau_{p}=P_{p}\cdot C_{C}\cdot d_{p}^{2}/18\cdot \mu_{a}$$ (11)

## Results and Discussion

### Spatiotemporal variation of the PM2.5 and PM10 mass concentrations

#### Diurnal variation of PM2.5 and PM10 mass concentrations at different heights

**Fig 3:**
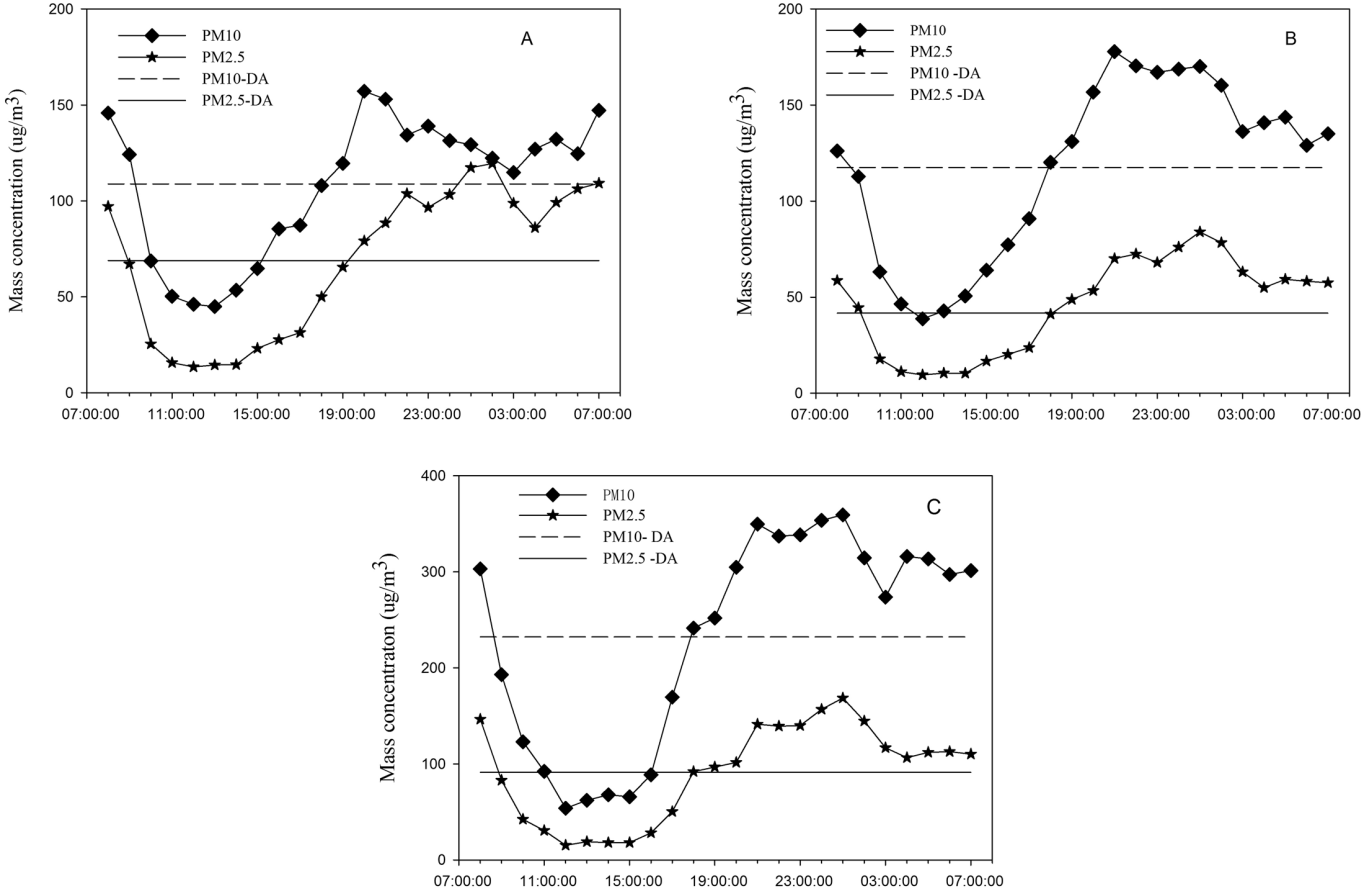
Daily variation of the particulate matter concentration. A is the daily variation at 1.5 m, B is that at 6 m, and C is that at 10 m, where DA is the daily average concentration of different particles.

Fig 3 shows the diurnal variation of the PM10 and PM2.5 mass concentrations at 1.5, 6, and 10 m. The trends for PM10 and PM2.5 were largely identical at the three different heights. PM10 and PM2.5 showed the highest value at about 21:00 pm and the average concentrations of PM10 at the heights of 1.5, 6, and 10 m are 157.19, 177.83, and 359.43 μg/m^3^, respectively. The average concentrations of PM2.5 at the heights of 1.5, 6, and 10 m are 119.5, 83.95, and 168.69 μg/m^3^, respectively. The lowest concentrations of PM10 and PM2.5 occurred at about 12:00 am, with that of PM10 at the heights of 1.5, 6, and 10 m being approximately 45.0, 38.72, and 55.5 μg/m^3^, respectively, and that of PM2.5 at the heights of 1.5, 6, and 10 m being approximately 13.51, 9.52, and 16.56 μg/m^3^, respectively. A high concentration of PM presented from dusk until the next morning, because a temperature inversion occurred in the surface layer in the morning and the atmosphere was stable, which caused the particles to accumulate at a high concentration [21]. Another reason was the low wind speed, low temperature, and slow vertical turbulence, which together caused slow particle diffusion and an increase in the PM concentration [22]. Besides, the wetland has a high relative humidity in the morning and night; at that time, the vapor has a high density, easily forming secondary aerosols [23]. At noon, the water vapor gradually descended to the lake or condensed, and the humidity gradually decreased. The particulate matter in the air settled, following the water vapor, and became dust [13]. From Fig 3, we can also conclude that the average concentration of PM10 and PM2.5 is different at the three heights. The trend in the average PM10 concentration at the three heights is 10 m > 6 m > 1.5 m, while that for PM2.5 is 10 m > 1.5 m > 6 m. The cause of this phenomenon was that the source of the PM2.5 is complicated and the dust storm on the surface had a significant impact on the PM2.5 concentration [24]. The average concentration of PM10 was the lowest at 1.5 m, as PM10 has a large diameter and is easily deposited on the water surface by gravity [25].

#### Periodic variation of the PM2.5 and PM10 mass concentrations at different heights

**Fig 4:**
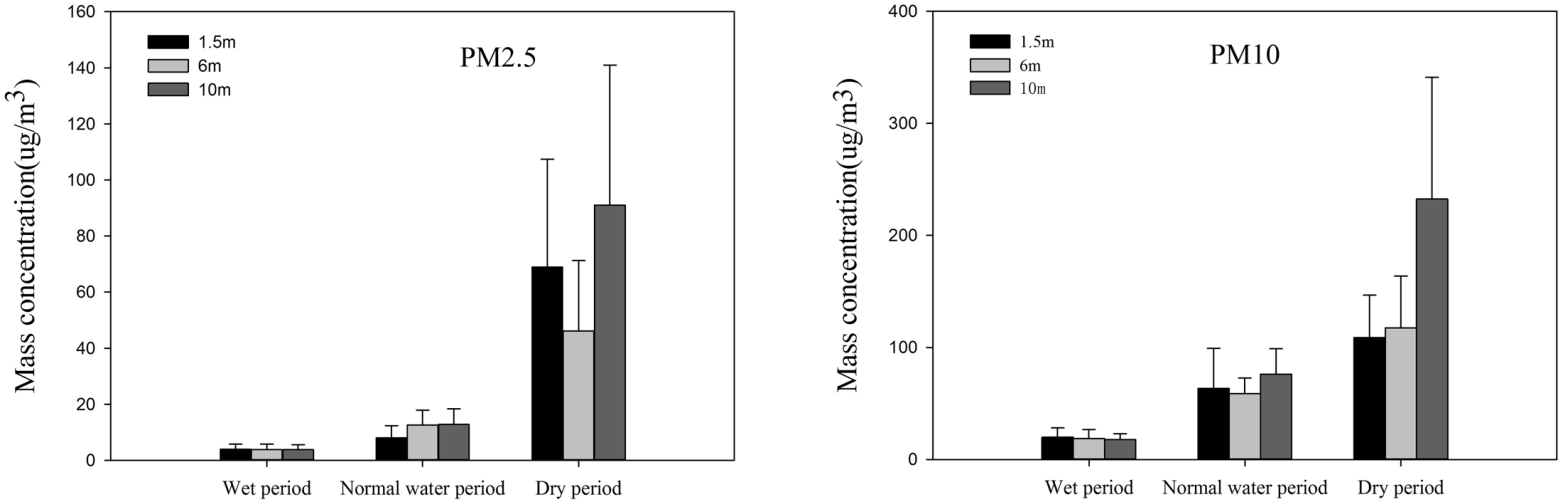
Changes in the particle concentration at different heights during different periods.

The year was divided into three periods according to the amount of water, including the dry period (winter), the normal water period (spring and autumn), and the wet period (summer). As shown in Fig 4, the periodic change in the concentrations of PM10 and PM2.5 at the three heights exhibited the same trend. The lowest value occurred during the wet period, followed by the value for the normal water period. However, the concentration during the dry period was the highest, being significantly higher than during the normal and wet periods. The result of this phenomenon was that the water froze during the dry period or at low relative humidity, so that dry deposition of the particulate matter was inhibited, and the absorption ability of plants, which withered, was reduced. In the wet period, the high relative humidity and lush vegetation can accelerate the dry deposition of particles, and thus the particulate matter reached its lowest level [11]. In addition, the high concentration of PM2.5 during the dry period was caused by anthropogenic emissions, primarily from coal combustion [26]. Ye et al. (2003), who studied the concentration and chemical composition of PM2.5 in Shanghai, also found that the PM2.5 concentration was highest in the winter and lowest in the summer [27]. Many former studies also indicated the same trends [28–29].

The concentrations of PM10 and PM2.5 at different heights showed some disparity during different periods. The lowest concentration of PM2.5 occurred at 6 m and the highest occurred at 10 m during the dry period, while the lowest concentration of PM10 occurred at 1.5 m and the highest at 10 m, which indicates that the wetland could also be a source of particles, especially PM2.5, during the dry period [30]. The lowest PM2.5 concentration occurred at 1.5 m and the highest occurred at 10 m during the normal water period, while the lowest PM10 concentration occurred at 6 m and the highest at 10 m. This situation was mainly due to meteorological factors and regional conditions [31]. During the wet period, PM10 and PM2.5 exhibited the same trend at the three heights, the lowest concentration occurring at 10 m and the highest occurring at 1.5 m, while the difference between the three heights was small. The trend during this period was significantly different to that for the other two periods, as the high relative humidity and low wind is unfavorable for diffusion and accumulation on the surface layer [32–33].

In order to understand the impact of time and space on the particulate matter concentration, an ANOVA was performed. The result indicated that the height and period both had an influence on the particulate matter concentration. There is no obvious difference in the particulate matter concentration at different heights, and the ANOVA results demonstrated that the p values for PM10 and PM2.5 are p = 0.717 and p = 0.815, respectively. While the particulate matter concentration differed significantly during different periods, the ANOVA results showed that the p values for PM10 and PM2.5 were p = 0.017 and p = 0.002, respectively. From these results, we can conclude that the height and period both have an influence on the particulate matter concentration, while the periodic influence on the particulate matter is more significant.

### Spatiotemporal variation of the chemical composition of PM2.5

#### The chemical composition and temporal variation of PM2.5

The water-soluble ions of PM2.5, Na^+^, NH_4_^+^, K^+^, Mg^2+^, Ca^2+^, SO_4_^2−^, NO_3_^−^, Cl^−^, HCOO^−^, and F^−^, were measured in this experiment. As listed in Table 1, the annual average SO_4_^2−^ concentration was 26.75 ± 4.25 μg/m^3^, constituting 42.22% of the water-soluble ions. Sulfate alone exceeded the US NAAQS annual PM2.5 standard of 15 μg/m^3^ [34]. The annual average NO_3_^−^ concentration was 7.98 ± 0.37 μg/m^3^, which accounted for 12.6% of the total ions. The annual average Cl^−^ concentration was 7.95 ± 0.52 μg/m^3^, accounting for 12.56% of the total water soluble ions. The sum of HCOO^−^, F^−^, Na^+^, NH_4_^+^, K^+^, Mg^2+^, and Ca^2+^ accounted for 32.62% of the total water-soluble ions. In conclusion, SO_4_^2−^, NO_3_^−^, and Cl^−^ were the dominant constituents of PM2.5. Our results were broadly consistent with many former studies [34–35]. Hu et al. (2002) analyzed the seasonal variation of ion species in fine particles at Qingdao and found that nitrate and ammonium, secondary formed compounds, are major components, in total accounting for 50% of the PM2.5 mass concentration. Yao et al. (2002) studied the water-soluble ionic composition of PM2.5 in Shanghai and Beijing, also finding that SO_4_^2−,^ NO_3_^−^, and NH_4_^+^ were the dominant ionic species.

**Table 1:** The annual and periodic mean concentrations of water-soluble ions in the wetland (μg/m^3^).

Ion	Normal water period	Wet period	Dry period	Annual
F^−^	0.11 ± 0.01	0.38 ± 0.08	0.36 ± 0.13	0.28 ± 0.07
Cl^−^	4.11 ± 0.47	9.94 ± 0.63	9.82 ± 0.46	7.95 ± 0.52
HCOO^−^	4.03 ± 0.71	1.12 ± 0.49	1.26 ± 0.89	2.14 ± 0.70
NO_3_^−^	10.70 ± 1.25	7.78 ± 1.36	5.46 ± 0.20	7.98 ± 0.94
SO_4_^2−^	23.60 ± 4.32	26.68 ± 4.36	29.97 ± 4.07	26.75 ± 4.25
Na^+^	2.46 ± 0.14	7.43 ± 0.77	2.39 ± 1.18	4.09 ± 0.70
K^+^	1.69 ± 0.36	1.12 ± 0.33	0.97 ± 0.15	1.25 ± 0.28
NH_4_^+^	4.46 ± 0.26	1.69 ± 0.14	3.05 ± 0.95	3.07 ± 0.45
Mg^2+^	0.66 ± 0.11	0.38 ± 0.07	0.28 ± 0.02	0.44 ± 0.06
Ca^2+^	3.77 ± 0.79	1.51 ± 0.40	1.28 ± 0.06	2.07 ± 0.42

Table 1 also shows that there were different characteristics for different ions during different periods. The highest concentration of SO_4_^2−^ occurred during the dry period during the whole experimental period. The concentration of SO_4_^2−^ during the wet period was higher than during the normal water period. This phenomenon can be attributed to a higher concentration of SO_2_ due to a higher rate of coal burning, combined with poor dispersion and a lower rate of removal for wet deposition. However, the concentration during the wet period was higher than during the normal period, due to secondary formation [26]. During the normal period, the concentration of NO_3_^−^ is much higher than during the wet period, as low temperatures are favorable for the formation of NO_3_^−^ [36]. However, the lowest NO_3_^−^ concentration occurred during the dry period, which was inconsistent with many other studies indicating that the NO_3_^−^ concentration in the winter was the highest [37]. The Cl^−^ concentration was higher during the dry period than in the wet and normal water periods, which is believed to be associated with coal burning in winter [34]. The NH_4_^+^ concentration during the normal water and dry periods is higher, indicating that high temperatures are not favorable for the conversion from NH_3_ to NH_4_^+^ [38]. Ca^2+^ and K^+^ were distinctly higher during the normal water period than during the wet and dry periods, which were reasonably attributed to the high mineral dust levels in the spring and the dry environment being favorable for soil resuspension [26, 39].

#### Spatial variation of the chemical composition of PM2.5

**Fig 5:**
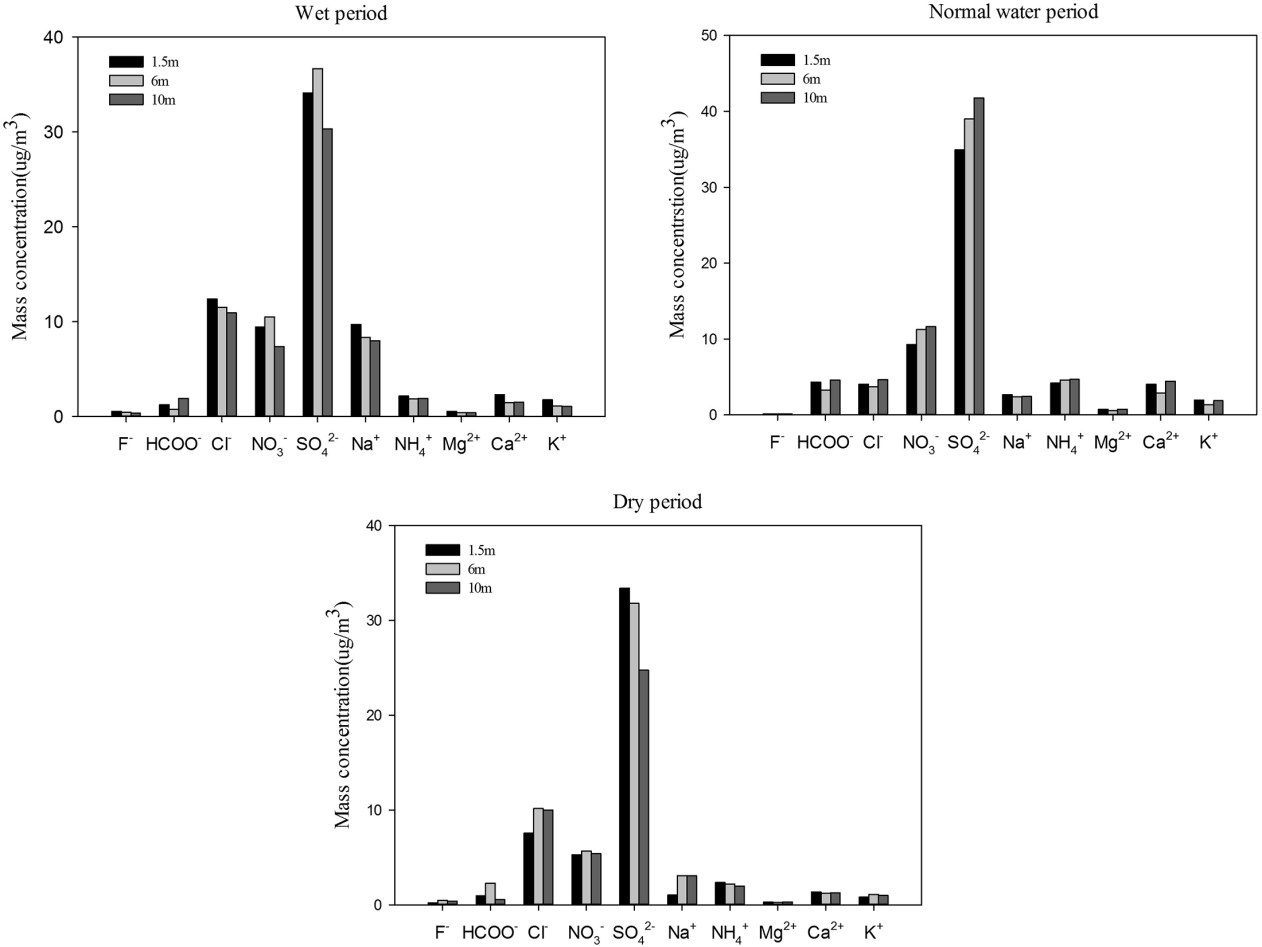
The chemical composition variation at different heights during different periods.

Changes in the chemical composition of PM2.5 at different heights are shown in Fig 5. During the wet period, the concentrations of SO_4_^2−^ and NO_3_^−^ exhibited the same trend. The highest value occurred at 6 m and the lowest at 10 m, because the PM25 concentration was the lowest at 10 m during this period. Cl^−,^ NH_4_^+^, and Na^+^ also showed consistent trends, in which the highest value occurred at 1.5 m and the lowest at 10 m. This result was completely consistent with the PM2.5 concentration trend at different heights. It can be shown that the PM2.5 and ion concentrations had a significant correlation during this period. During the normal water period, the ions, SO_4_^2−^, NO_3_^−^, and NH_4_^+^ exhibited the same trend as the PM2.5 concentration. The highest values for SO_4_^2−^ and NH_4_^+^ occurred at 1.5 m and the lowest values at 10 m during the dry period. The highest values for Cl^−^ and NO_3_^−^ occurred at 6 m and the lowest values at 1.5 m.

### Dry deposition velocity

#### The dry deposition velocity of PM2.5 and PM10 during different periods

The PM2.5 and PM10 deposition velocity during different periods is summarized in Table 2. An ANOVA was performed and the p values were calculated to show the differences during different periods. The PM2.5 deposition velocity during the dry period (1.50 ± 0.79 cm s^−1^) was significantly higher than that during the wet period (0.08 ± 0.03 cm s^−1^, p = 0.009) and the normal water period (0.16 ± 0.01 cm s^−^1, p = 0.01); however, the deposition velocity did not differ significantly between the wet and normal water periods (p = 0.825). Moreover, the PM10 deposition velocity during the dry period (4.88 ± 1.22 cm s^−1^) was also significantly higher than during the wet period (1.56 ± 0.35 cm s^−1^, p = 0.002) and the normal water period (4.88 ± 1.22 cm s^−1^, p = 0.02), and that during the normal water period was significantly higher than during the wet period (p = 0.05). This result could be attributed to the influence of meteorological factors on the particle deposition velocity; the higher relative humidity and the stronger wind speed in the summer, compared to other seasons in the wetland, may explain why the lowest deposition velocity is recorded during the wet period [12–13].

**Table 2:** Dry deposition velocity of PM2.5 and PM10 during different periods.

Particulate matter	Periods	Deposition velocity (cm s^−1^)
PM2.5	Normal water period	0.16 ± 0.01
	Wet period	0.08 ± 0.03
	Dry period	1.50 ± 0.79
PM10	Normal water period	4.88 ± 1.22
	Wet period	1.56 ± 0.35
	Dry period	9.17 ± 2.56

#### The dry deposition velocity of PM10 and PM2.5 during the daytime and the night-time

As shown in Table 3, the deposition velocity of PM2.5 and PM10 during the daytime and the night-time was summarized. The PM2.5 average deposition velocity decreased during the daytime (0.33 ± 0.19 cm s^−1^), whereas it increased during the night-time (0.83 ± 0.20 cm s^−1^). The PM10 average deposition velocity in the daytime (4.09 ± 1.57 cm s^−1^) was also lower than during the night-time (6.42 ± 0.76 cm s^−1^). This result was different from previous studies, in which the deposition during the daytime was higher than during the night-time [40]. This is mainly due to the high relative humidity during the night-time in the wetland.

**Table 3:** Dry deposition velocity of PM2.5 and PM10 during the daytime and night-time.

Periods	Times	Dry deposition velocity(cm s^−1^)	
		PM2.5	PM10
Normal water period	Daytime	0.13 ± 0.02	3.62 ± 1.44
	Night-time	0.19 ± 0.01	6.20 ± 0.82
Wet period	Daytime	0.06 ± 0.03	1.40 ± 0.37
	Night-time	0.09 ± 0.02	1.68 ± 0.20
Dry period	Daytime	0.80 ± 0.53	6.96 ± 2.91
	Night-time	2.20 ± 0.57	11.39 ± 1.25

### Dry deposition flux

#### PM dry deposition flux at different heights during different periods

**Fig 6:**
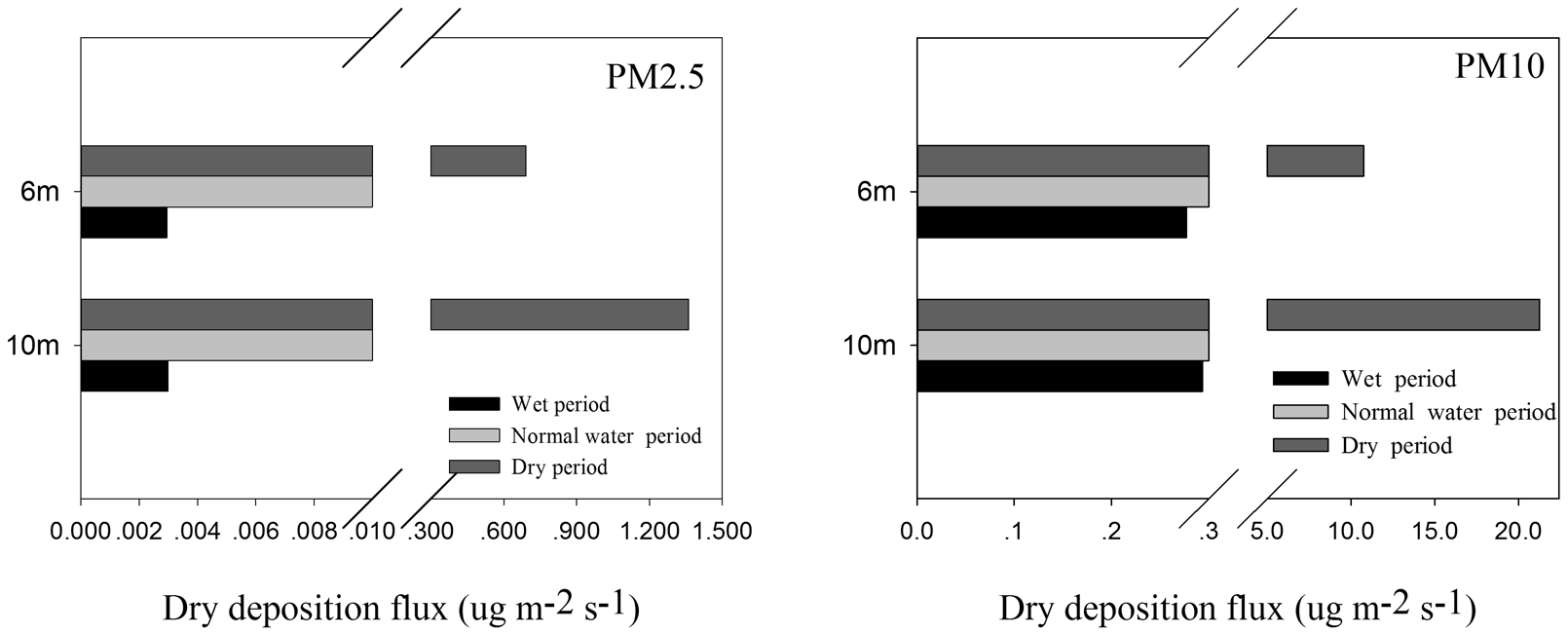
The PM2.5 and PM10 dry deposition flux at different heights during different periods.

The PM2.5 and PM10 dry deposition flux at different heights during different periods was calculated using the dry deposition model, and the result is shown in Fig 6. The PM2.5 average dry deposition flux during different periods was 0.46 μg m^−2^ s^−1^ at 10 m and 0.24 μg m^−2^ s^−1^ at 6 m. The dry deposition at 10 m was higher than at 6 m, but the difference between the two values was not significant (p = 0.68). For PM10, the average dry deposition during different periods was 8.37 μg m^−2^ s^−1^ at 10 m and 4.62 μg m^−2^ s^−1^ at 6 m. The dry deposition at 10 m was higher than at 6 m, but the difference between the two values was also insignificant (p = 0.63). The PM deposition was significantly correlated with the PM concentration [41]. The PM concentration during different periods at 6 and 10 m was not significantly different (p = 0.65 for PM2.5 and p = 0.57 for PM10), and the difference between the PM deposition flux at 6 and 10 m also turned out to be insignificant.

The PM dry deposition flux during different periods is also shown in Fig 6. The PM2.5 dry deposition flux during the dry period (1.03 μg m^−2^ s^−1^) was significantly higher than in the wet period (0.003 μg m^−2^ s^−1^, p = 0.033) and in the normal water period (0.02 μg m^−2^ s^−1^, p = 0.035). Also, the PM10 dry deposition flux during the dry period (16.01 μg m^−2^ s^−1^) was significantly higher than during the wet period (0.29 μg m^−2^ s^−1^, p = 0.035), but the difference between the dry and normal water periods (3.2 μg m^−2^ s^−1^, p = 0.06) was insignificant. Because the PM concentration has an impact on the PM dry deposition, the concentration during the dry period was higher than during the other periods. Another reason for this was that the deposition velocity during the dry period was higher than during the other periods. Therefore, the dry deposition flux during the dry period turned out to be the highest.

#### PM dry deposition flux during the daytime and night-time

**Fig 7:**
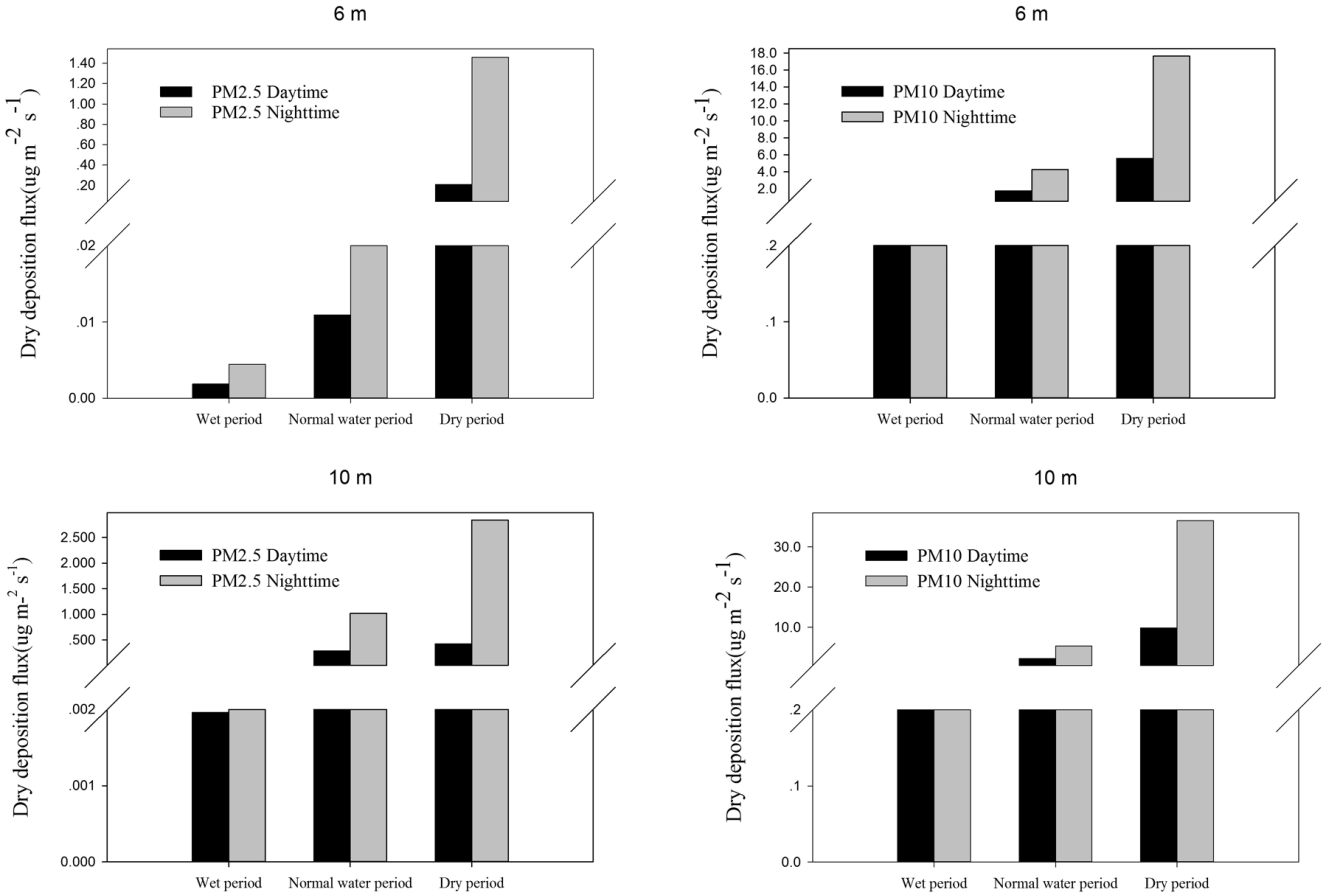
The PM2.5 and PM10 dry deposition flux during the daytime and the night-time.

Fig 7 shows the PM2.5 and PM10 dry deposition flux during the daytime and night-time. Generally, the PM2.5 and PM10 dry deposition flux decreased during the daytime and increased during the night-time at different heights during different periods. These results are due to the deposition velocity during the night-time being higher than in the daytime and the average concentration also being higher during the night-time than during the daytime. Besides, specific meteorological factors have an impact on the dry deposition flux. By performing a correlation analysis, we can conclude that the PM2.5 dry deposition flux was negatively correlated with the temperature and wind speed (r = −0.80 and −0.25) and positively correlated with the relative humidity (r = 0.12). The PM10 dry deposition flux was also negatively correlated with the temperature and wind speed (r = −0.88 and −0.17, respectively) and positively correlated with the relative humidity (r = 0.2). Moreover, the relationship between the PM dry deposition flux and the meteorological factors was almost constant at different heights.

Fig 7 shows the PM2.5 and PM10 dry deposition flux during the daytime and night-time. Generally, the PM2.5 and PM10 dry deposition flux decreased during the daytime and increased during the night-time at different heights during different periods. These results are due to the deposition velocity during the night-time being higher than in the daytime and the average concentration also being higher during the night-time than during the daytime. Besides, specific meteorological factors have an impact on the dry deposition flux. By performing a correlation analysis, we can conclude that the PM2.5 dry deposition flux was negatively correlated with the temperature and wind speed (r = −0.80 and −0.25) and positively correlated with the relative humidity (r = 0.12). The PM10 dry deposition flux was also negatively correlated with the temperature and wind speed (r = −0.88 and −0.17, respectively) and positively correlated with the relative humidity (r = 0.2). Moreover, the relationship between the PM dry deposition flux and the meteorological factors was almost constant at different heights.

### Relationship between meteorological factors and particulate matter concentrations

**Fig 8:**
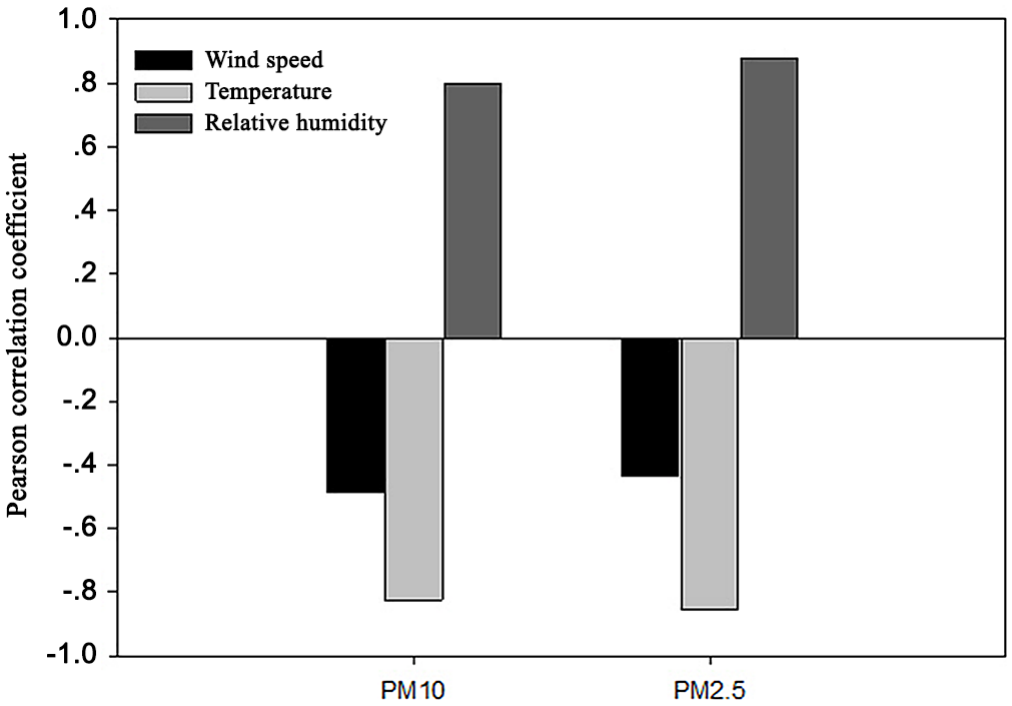
Correlation between the particulate matter concentration and meteorological factors.

The former studies documented that the meteorological factors strongly influenced the concentrations of PM2.5 and PM10. Peng et al. (2011) found that the wind could control the accumulation and dispersion of the particles [42]. Fig 8 shows the correlations between the PM and several meteorological variables, indicating that the PM2.5 and PM10 concentrations had a significant correlation with the wind speed, temperature, and relative humidity (confidence level is 99%). The PM concentrations in the wetland were negatively correlated with the wind speed and temperature and positively correlated with the relative humidity because as the temperature and wind speed increases, the capacity for diffusion also increases [43–44]. In addition, PM2.5 appears to be more sensitive to meteorological factors. These results were determined by former studies [18].

**Fig 9:**
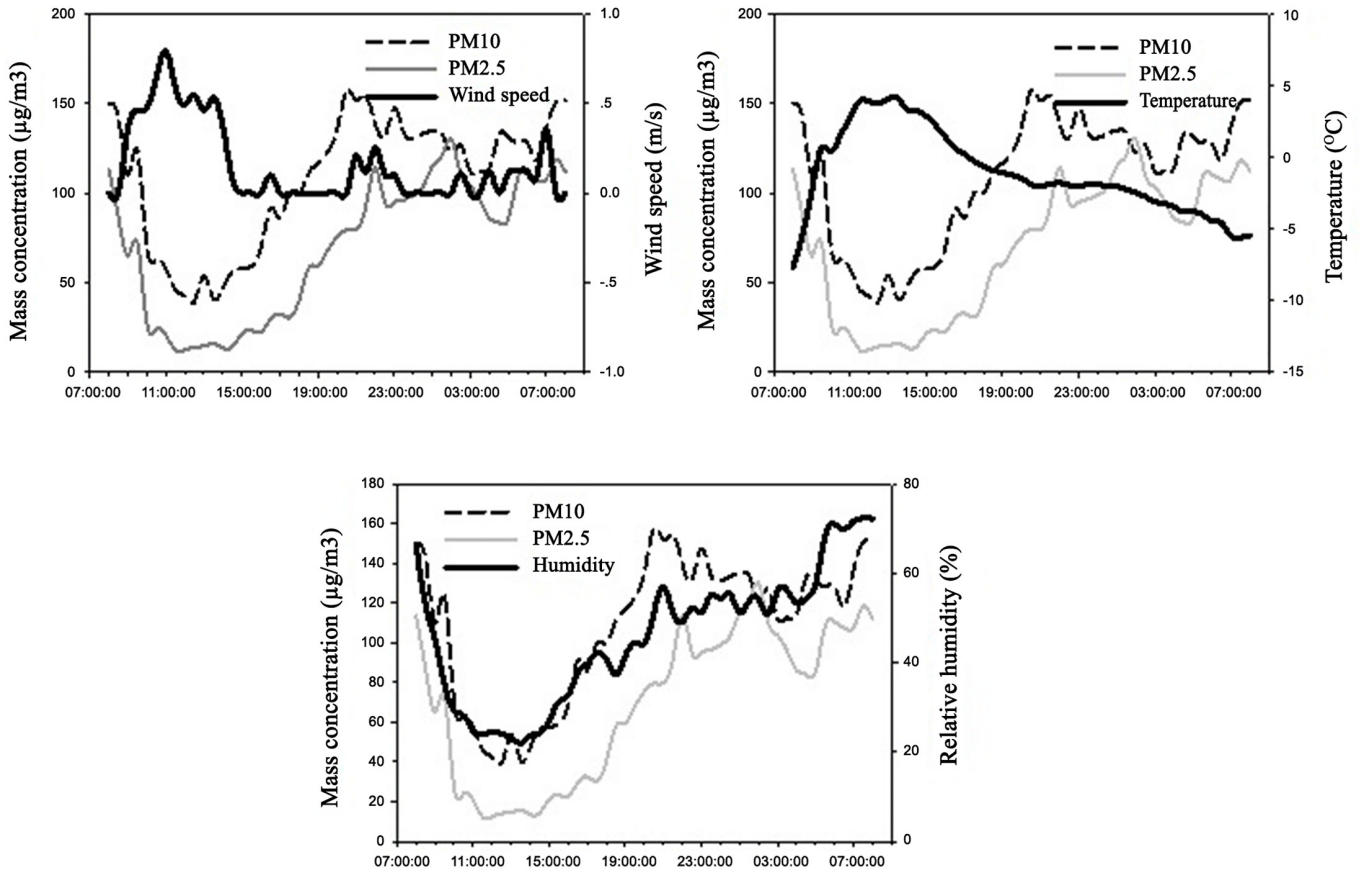
The relationship between meteorological factors and the particulate matter concentration.

The detailed relationship between meteorological factors and the particulate matter concentration is shown in Fig 9. The PM2.5 and PM10 concentrations were negatively correlated with the wind speed. The PM2.5 and PM10 concentrations have a tendency to increase as the wind speed decreases, and a tendency to decrease as the wind speed increases, as a low wind speed was unfavorable for particle diffusion but favorable for the accumulation of particles in the wetland [23]. The PM2.5 and PM10 mass concentrations were significantly negatively correlated with temperature. The temperature gradually increased and reached a peak at about 11:00 am, and the PM2.5 and PM10 concentrations dropped to their lowest values, which was due to the increasing temperature on the ground at noon being favorable for atmospheric motion and thus for particle diffusion. When the temperature is relatively low in the morning and afternoon, or during atmospheric inversions, the atmosphere is stable, which is not favorable for particle diffusion, and thus the concentration is high.

The relative humidity was significantly positively correlated with the PM2.5 and PM10 concentrations. When the humidity was high, the particles grew too heavy to stay in the air, and thus the concentration increased [45].

## Conclusion

In this study, from 2014 to 2015, we have investigated particulate matter concentrations, chemical compositions, the dry deposition flux, and meteorological factors at Cuihu wetland park.

The diurnal variations of PM10 and PM2.5 were largely identical at the three different heights. The highest concentration occurred at 21:00 pm, with the PM10 concentration at the heights of 1.5, 6, and 10 m being 157.19, 177.83, and 359.43 μg/m^3^, respectively, and the PM2.5 concentration at heights of 1.5, 6, and 10 m being 119.5, 83.95, and 168.69 μg/m^3^, respectively. The lowest concentration occurred at about 12:00 am, with that of PM10 at the heights of 1.5, 6, and 10 m being approximately 45.0, 38.72, and 55.5 μg/m^3^, respectively, and that of PM2.5 at the heights of 1.5, 6, and 10 m being approximately 13.51, 9.52, and 16.56 μg/m^3^, respectively. Over the year, the lowest concentrations of PM10 and PM2.5 occurred during the wet period, followed by those during the water normal period. In addition, the concentration during dry period was largely higher than in other period. However, the concentration trends at different heights showed some disparity.

SO_4_^2−^, NO_3_^−^, and Cl^−^ were the dominant components of PM2.5, accounting for 42.22, 12.6, and 21.56%, respectively. In addition, the SO_4_^2−^ concentration was higher during the dry and wet periods than during the normal water period, and the concentration at 6 m was the highest. The NO_3_^−^ concentration during the normal water period was higher than during the wet and dry periods, and the concentration was highest at 6 m. During the day, the Cl^−^ concentration was higher than during other periods, and the highest value occurred at 1.5 m.

The PM2.5 and PM10 deposition flux at 10 m was higher than at 6 m during the sampling periods and the deposition flux during the dry period (1.03 μg m^−2^ s^−1^ for PM2.5 and 16.01 μg m^−2^ s^−1^ for PM10) was higher than during the wet period (0.003 μg m^−2^ s^−1^ for PM2.5 0.29 μg m^−2^ s^−1^ for PM10) and the normal water period (0.02 μg m^−2^ s^−1^ for PM2.5 and 3.2 μg m^−2^ s^−1^ for PM10). Besides, the deposition flux during the daytime was lower than during in the nighttime. Moreover, meteorological factors affected the deposition flux; the temperature and wind speed were negatively correlated with the deposition flux and the humidity was positively correlated.

The meteorological factors significantly affected the particulate matter concentration; the temperature and wind speed were negatively correlated, and the relative humidity was positively correlated with the PM2.5 and PM10 concentrations.
